# Anything You Can Do, I Can Do Better: Can Aptamers Replace Antibodies in Clinical Diagnostic Applications?

**DOI:** 10.3390/molecules24234377

**Published:** 2019-11-30

**Authors:** Michelle Bauer, Mia Strom, David S Hammond, Sarah Shigdar

**Affiliations:** 1 School of Medicine Deakin University, Geelong, Victoria 3128, Australia; mbauer@deakin.edu.au (M.B.); mfstrom@deakin.edu.au (M.S.); david.hammond@deakin.edu.au (D.S.H.); 2 Centre for Molecular and Medical Research, Deakin University, Geelong, Victoria 3128, Australia

**Keywords:** aptamers, antibodies, clinical, diagnostics, immunohistochemistry, immunophenotyping, lateral flow devices

## Abstract

The mainstay of clinical diagnostics is the use of specialised ligands that can recognise specific biomarkers relating to pathological changes. While protein antibodies have been utilised in these assays for the last 40 years, they have proven to be unreliable due to a number of reasons. The search for the ‘perfect’ targeting ligand or molecular probe has been slow, though the description of chemical antibodies, also known as aptamers, nearly 30 years ago suggested a replacement reagent. However, uptake has been slow to progress into the clinical environment. In this review, we discuss the issues associated with antibodies and describe some of the applications of aptamers that have relevancy to the clinical diagnostic environment.

## 1. Introduction

In the age of the reproducibility crisis, researchers are continuously searching for validated molecular probes that provide robust assays, tests that simply work reliably not only in their own laboratories, but also when utilised by others [1,2,3]. An estimated 20% of reproducibility failures are due to variability of standard antibody reagents, though this number is likely much higher [2,4]. But how are antibodies introducing variability into assays, confounding results, and stifling reliable replication?

Whilst concerns regarding reproducibility are nothing new, and the causes most certainly multifactorial, concern over the role that research-grade antibodies play in the reproducibility crisis is garnering attention [2,3,4,5,6,7]. Reagent variations account for an estimated 36% of total irreproducibility [8] of biological assays, with antibodies representing the most ubiquitously utilised group of reagents [3]. Research-grade antibodies are big business; there are currently around 3.8 million research antibodies [9] marketed by over 300 different companies [2], with well-known variability between vendors when it comes to efficacy. A 2008 validation survey conducted by the Human Protein Atlas [10] assessed more than 5000 commercial antibodies from 51 different vendors utilising Western blot and immunohistochemistry on fixed-tissue microarrays. Astoundingly, results showed that only 49% could be successfully validated. Furthermore, when stratified by vendor, success rates for antibody validation showed immense variability between suppliers (range of 0 to 100%) [10]. Reagents portal antibodies-online.com reports similar findings, with less than 50% of research antibodies making the grade when subjected to independent validation [2].

The ongoing problem of diagnostic antibody variability is highlighted well by a recent paper which tested 16 commercial antibodies (from seven different vendors) to C9ORF72, a protein specific to amyotrophic lateral sclerosis (ALS) [11]. Notable findings were that only one antibody worked accurately in immunofluorescent applications, with a further two showing strong specific signals via Western blot. In addition to the poor rate of validation success, the implication of this result is that multiple antibodies specific for each application to which they are applied are required (immunofluorescence vs. Western blot), adding further layers of complexity and cost to experimental protocols. Of higher concern, these findings relay that these antibodies, which have been cited in multiple publications, failed validation by this research group, meaning the results from such studies should be interpreted with caution and/or disregarded.

Extrapolating antibody validation failure data across the scientific community, Bradbury and Plückthun (2015) estimate that around half of the 1.6 billion USD spent globally on research antibodies each year is “money down the drain” [5]. Contributing factors to this situation are an oversupplied antibody vendor market, with extensive selling/rebranding of reagents coupled with substandard reporting of research materials in the literature [4]. These factors often culminate in the inability to correctly identify the original antibody reagent from publications by vendor and catalogue number—let alone batch number—meaning the quality control data is unattainable and accurate replication is not possible. As a result, and frustratingly for many in the scientific community, verifying and obtaining the same antibody and reproducing similar binding effectiveness is ‘nigh impossible’ even when the batch number is known [2]. Batch-to-batch inconsistencies add a further confounder in terms of reproducibility [12]. The potential for cross-reactivity and lack of consistency between batches of polyclonal antisera is well-known. Almost all researchers who routinely use antibodies have a tale of variation between different lot numbers of the same antibody. This is largely due to the fact that only around 0.5% to 5% of total antibodies in a polyclonal reagent are actually specific for the cognate target [5]. Additionally, affinity purification of animal sera is not always sufficient to remove all cross-reactive clones [13]. Therefore, there is significant batch-to-batch variation—even when the very same animal is re-immunised [13]. Batches originating from a new generation of animal are less consistent still. Yet, even in this case, some vendors are not compelled to assign a new batch number to the existing reagent [2,6,7,14]. The ultimate result from the shortcoming of antibody reagent variations is difficulty in verifying and building upon the body of previously published work in any field of the biological sciences, delaying the progress of discoveries.

When a rigorously validated monoclonal antibody is available, it is important to remember that application-specific validation is still required to assess functionality and target accessibility. Furthermore, recent evidence suggests that repeated validation may still be required with each new batch. Lot-to-lot variability with monoclonal antibodies is more frequent than the wider scientific community would assume [3,5,12,15,16]. It is apparent that some hybridoma clones may be propagated for decades. Genetic drift is inevitable with repeated passaging over such time frames but, in some cases, such reagents are still accompanied by the validation data from the original batch [2]. Since monoclonal antibodies are rarely characterised to the level of their sequence information, mutation and even loss of antibody genes over time may result in the loss of a reagent that may have been well-characterised (at high cost) and crucial to a particular body of research. Furthermore, some monoclonal antibody preparations are, in reality, oligoclonal—and the dominant clonal species may change with repeated passaging [13]. Moreover, some monoclonal antibodies are still propagated in vivo in order to produce commercially relevant yields, and these preparations may become contaminated by non-specific immunoglobulins from the ascites fluid of the host animal [17]. As a result of such inconsistencies, some experts advocate bulk purchasing from a single validated batch to ensure the consistency of data generated during long term projects [14]. However, others warn of the downside to such hoarding practices, with loss of functionality seen with some antibody clones in as little as 4–5 months of responsible storage [12]. Subtle lot-to-lot variation with monoclonal antibodies becomes more evident in methods attempting to quantify low-abundance targets—requiring high-sensitivity affinity-capture reagents, such as chromatin immunoprecipitation (ChIP) for the study of epigenetic modifications.

## 2. Working Toward a ‘Perfect’ Molecular Probe 

A key consideration when maintaining reproducibility of results, particularly for longitudinal studies or clinical diagnostic development, is the uninterrupted supply of affinity reagents of consistent specificity and sensitivity to the intended target(s) of interest [5,7]. The precarious nature of some affinity reagents can ultimately lead to disaster. More specifically, when a new batch fails to recapitulate past results and/or when supply is interrupted or halted without warning [18], the speed of research progress is impeded. Furthermore, not all companies are immediately upfront in confirming the discontinuation of an antibody [2,19], which may leave long-term projects in limbo, resulting in further wastage of time and money. Ultimately, such nonchalance may culminate in ongoing irreproducible and/or confounded conclusions when conducting studies, resulting in the waste of sometimes years of hard work.

In response, Bradbury and Plückthun (along with more than 100 co-signatories) have proposed to initiate a transition towards ‘renewable’ affinity reagents—including molecules such as recombinant antibodies, protein scaffolds (affibodies), and aptamers—which are specified at the molecular sequence level [5,18].

The premise of sequence-defined recombinant antibodies may circumvent much of the batch variability introduced by production in animals [2]. The methodology also represents a comfortingly familiar option for companies that have invested millions in the production, marketing, and humanisation of monoclonal antibodies [20]. However, sequence-defined recombinant antibodies do not yet represent a cost-effective alternative to conventional monoclonal antibodies [7,20]. Unavoidable variabilities in recombinant antibody generation also still exist, such as subtle batch-to-batch variabilities associated with cell-based cloning [18]. The rational conclusion of many in the scientific community is that, even with extensive research, recombinant monoclonal antibodies have not, nor are likely to, fulfil the role of the ‘perfect’ molecular probe. The issues of irreproducibility, cross-reactivity, and poor validation, innate to monoclonal antibody production, have led to this conclusion. In addition, as antibodies are polypeptides, they have an intrinsically shorter half-life—suffering from degradation over time, even with responsible storage and handling, ultimately affecting their reliability. Ensuring the antibody used in the experimental system is still viable is one of the main reasons that controls are included in every assay [21].

Identifying and understanding these issues is the starting point for defining the key properties of the ‘perfect’ molecular probe. The ideal molecular probe would be a molecule that is highly reproducible in its binding sensitivity and specificity (with limited cross-reactivity) and also highly stable in structure, affording a long half-life. A candidate group of molecules that appear to possess these qualities are oligonucleotide aptamers.

## 3. Aptamers: The Chemical ‘Antibodies’

Aptamers were first described in 1990 and are colloquially known as ‘chemical antibodies’ [22,23,24] to distinguish them from their protein antibody counterparts. This term was coined due to the similarity between antibodies and aptamers in that they bind specifically with their target via an induced fit mechanism [25,26]. With a backbone of DNA or RNA, intra-nucleotide binding contributes to the tertiary conformation of aptamers, conferring the ability to specifically bind to complimentary molecules, whether they are protein or nucleic acid in nature. Aptamers are, by their very nature, sequence-defined and are chemically synthesised, and therefore represent a cost-effective, highly consistent, and easily modifiable alternative to antibodies [27]. They also possess unique properties which are beyond the capabilities of protein-based reagents, as detailed quite eloquently by John Bruno a few years ago [20].

Aptamers are generated using a completely in vitro process known as Systematic Evolution of Ligands by EXponential enrichment (SELEX), which involves iterative cycles of incubation of a randomised library of nucleic acid sequences with the target of interest [23]. This high throughput screening process essentially represents a Darwinian ‘survival of the fittest’ evolution, whereby, the stringency of each cycle is increased and only sequences that bind with high sensitivity to the target are propagated to the next selection round. To ensure specificity of the bound sequences, negative selection steps with similar proteins or target molecules are included in the protocol. Aptamers can be generated to several isoforms of the same target protein by using a procedural variant known as ‘toggle’ SELEX, which switches proteins during the selection rounds to generate aptamers that bind to a consensus region [28].

The iterative nature coupled with the inherent stringency of the SELEX process results in the generation of aptamers which exhibit superior specificity when compared to equivalent target protein antibodies [29]. Once the sequence of the aptamer is known, it becomes essentially immortalised, allowing it to then be chemically synthesised, resulting in no batch-to-batch variation. This phenomenon fulfils one aspect of the ‘perfect molecular probe’ criteria and arguably confers aptamers their greatest advantage over conventional antibodies in that they can be synthesised with 100% reproducibility. Furthermore, when the aptamer probe sequence is specified in the published literature, it is easily transferrable to other researchers, enabling the scientific community to reliably build on the work of others in a step-wise fashion.

## 4. Diagnostic Applications for Aptamers

### 4.1. Immunohistochemistry

Within diagnostic pathology and clinical laboratories, outside of automated routine testing, are specialised tests requiring human interaction. Probably one of the largest areas is the histopathology laboratory, where diagnoses are made based on the assessment of patient tissue samples. In a typical diagnostic workflow, tissue is taken from a patient and stained with haematoxylin and eosin to distinguish different tissue structures. In the case of cancers, when a diagnosis cannot be made on the basis of tissue morphology alone, immunohistochemistry (IHC) may be performed to ascertain whether specific biomarkers are present or absent, guiding treatment decisions. Immunohistochemistry on fixed-tissue sections can take 24–48 h to yield a result under best-case conditions. If we consider the issues described above, where lack of antibody specificity and/or batch-to-batch variation can produce inconsistent results, it becomes clear that definitive diagnosis may be delayed at best or erroneous at worst, ultimately limiting or delaying treatment options [30,31].

**Figure 1:**
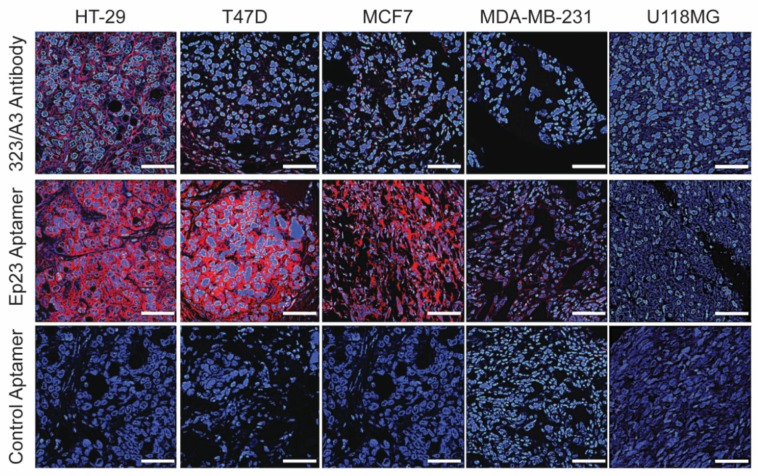
Sensitivity of antibodies and aptamers to EpCAM in different cell lines. Immunofluorescence staining of colon cancer (HT-29), breast cancer (T47D, MCF7, and MDA-MB-231), and glioblastoma (U118MG) xenograft tumors by EpCAM antibody, 323/A3, and EpCAM aptamer, Ep23, and control aptamer (blue: nuclei; red: EpCAM positive staining). Aptamer staining was performed for 15 min at 37 °C, while 323/A3 staining was performed at 4 °C overnight. The antibody is unable to distinguish between moderate and low expression of EpCAM whereas the aptamer staining intensity is relative to EpCAM expression. All fluorescent images were taken under a confocal microscope with 60× magnification. Images are representative of at least three separate experiments. Scale bar: 50 mm.

Several studies have compared aptamers to antibodies for the histochemical staining of both fresh-frozen and paraffin-embedded tissue sections [32,33,34,35]. These studies demonstrated that the aptamers presented much less background staining, a much more sensitive detection, and a shorter incubation period. This latter point—significantly shorter incubation times (15–20 min versus 1 h to overnight)—indicates that aptamers may also be useful for intra-operative procedures. Interestingly, aptamers appear to have a better analyte response curve and are able to discriminate between low, moderate, and high expression, whereas analyte sensitivity for antibodies can be quite limited (Figure 1) [36,37]. While some minor amendments to the existing IHC protocols may be necessary depending on the aptamer or the target biomarker, ultimately, aptamers can replace traditional antibodies in such applications with very limited capital expenditure, additional resources/reagents, or staff training being required. We have also tested the ability of aptamers to sequentially stain different biomarkers within the same cellular compartment and hypothesised that aptamers have potential for this, as their small size may limit steric hindrance (unpublished data), with preliminary results demonstrating superiority in this type of assay. Further reviews of the applications of aptamers in immunohistochemistry have been provided by Bauer et al. and Bukari et al. [35,38].

### 4.2. Immunophenotyping 

Immunophenotyping of blood cells, such as for the diagnosis of leukaemias, is another example of specialised testing performed by clinical laboratories which relies on the use of antibodies. Flow cytometry is an indispensable method for immunophenotyping applications. Aptamers offer a unique advantage over antibodies in flow cytometry applications, as almost any fluorophore can be conjugated to an aptamer with a 1:1 stoichiometric ratio of aptamer to fluorophore. In contrast, the only fluorophore that currently conjugates to an antibody in a 1:1 ratio is phycoerythrin, limiting the ability to run reliably quantitative assays of multiple cell surface markers on the same cell [39]. Aptamers therefore have the potential to be more efficiently used for the multiplexed quantitation of cell surface markers. It should be noted that part of the characterisation process for aptamer binding to native proteins involves flow cytometry to confirm specificity and sensitivity; therefore, this probably represents the most comprehensively validated protocol for aptamers [40,41,42].

### 4.3. Enzyme-Linked Sorbent Assays

While both previous applications of immunohistochemistry and flow cytometry have the benefits of human subjective interpretation, there are a number of assays that rely exclusively on automated readouts. As such, these tests require a high sensitivity and specificity to prevent false positives and false negatives. The enzyme-linked immunosorbent assay (ELISA) provides a familiar example, as it represents the mainstay of analytical biochemistry and may also replace indirect immunofluorescence for the detection of autoantibodies [43]. ELISAs are used as both a diagnostic tool and an assay for the purpose of discerning the concentration of analytes, particularly proteins within a solution of proteins [44]. There are several formats for ELISAs, including direct, indirect, and sandwich ELISA, with the commonality of a reliance on antibodies in each of these formats. Direct ELISAs utilise enzyme-labelled antibodies, while indirect ELISAs typically use a polyclonal-enzyme-labelled antibody that binds to the analyte-specific antibody, and sandwich ELISAs use a combination of both approaches [45]. While ELISAs represent a current gold standard method for clinical diagnostics, they suffer from the same disadvantages previously described when utilising antibodies as molecular probes.

**Figure 2:**
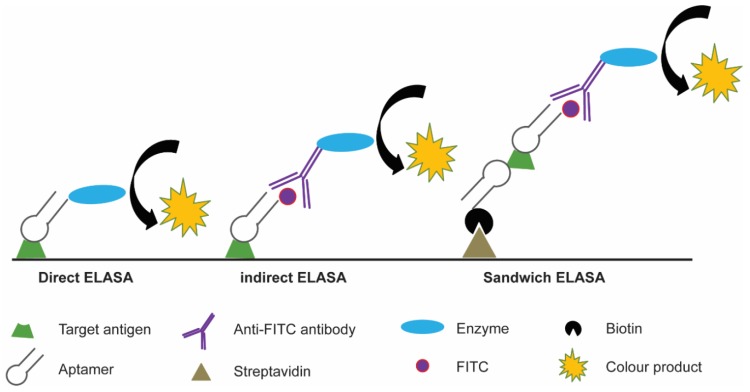
Schematic of enzyme-linked aptamer sorbent assay (ELASA). In direct ELASA, the aptamer can be conjugated to an enzyme or other reporter molecule directly; in an indirect ELASA, the aptamer is first labelled with a reporter molecule, such as FITC, to which a secondary antibody-enzyme conjugate binds; in the sandwich ELASA, a biotinylated aptamer is bound to a surface to capture the target antigen, and the same aptamer-reporter molecule and secondary antibody pairing are used to provide a colourimetric change.

Aptamers have been investigated as molecular probe replacements for antibodies in ELISA-like assays, with the acronym ‘ELASA’ coined (enzyme linked apta-sorbent assay). It should also be noted that these assays may also be known as ELONA (enzyme-linked oligonucleotide assay). Many ELASA configurations have been described, including direct, indirect and several sandwich ELASA formats, which utilise aptamers either alone or in combination with antibodies (Figure 2) [46,47,48,49]. The commonality in the findings of these studies was that they each demonstrated increased sensitivity and reduced limit of detection compared to their antibody counterparts. One example of an ELASA that has shown promise in patient-derived samples utilised the aptamer generated to secreted proteins from Mycobacterium tuberculosis [50]. When tested in serum samples from active pulmonary tuberculosis (TB) patients, extrapulmonary TB patients, and healthy donors, the ELASA showed a sensitivity and specificity of 100% and 94.1%, respectively. The utility of this assay has been further demonstrated in analysing sputum samples from TB patients, with a reported sensitivity and specificity of 91.3% and 90%, respectively [51]. To put this into perspective, analysis of commercial ELISA kits showed the highest sensitivity and specificity to be 83.3% and 98.9%, respectively [52]. It has only been by combining a cocktail of reagents that sensitivity and specificity can be increased above 98% [53].

While the majority of reported ELASAs to date have been developed for infectious agents [54,55], aptamer-based ELASAs have also been developed to assist in the early diagnosis of hepatocellular carcinoma. These ELASAs have also shown comparable results to the conventional ELISA in patient samples [56]. These examples allow one to conclude that, in equivalent applications, aptamer-based ELASAs demonstrate comparable, if not superior, sensitivity and specificity than existing ELISA methodologies. This situation once again demonstrates the benefits of aptamers compared to antibodies, and our learned opinion is that the lack of batch-to-batch variation, as well as room temperature storage stability, could well see aptamers replacing antibodies for related applications in the near future. Some interesting considerations were recently detailed in a paper looking at the effect that variables such as aptamer-target affinity and the density of aptamer loading onto the microplate-well surface has on ELASA sensitivity. Whilst the relatively small size of the aptamer is generally discussed as an advantage over antibodies, it can prove disadvantageous for certain applications. As detailed by Kimoto and colleagues, high affinity aptamers showed reduced signal intensity when higher aptamer concentrations were adsorbed to the surface of the ELASA plate, but this phenomenon was not observed with weaker binders. The authors postulated that the tight clustering of the high affinity aptamers might cause aptamer–aptamer interactions, which may hinder binding to their target proteins. When the binding density of the biotinylated aptamers to the microplate was reduced through the use of an anti-biotin IgG (~150kDa), rather than streptavidin (~53kDa), as the functionalised microplate surface, the signal intensities were greatly improved [57]. This correlates well with our own findings in similar assays (unpublished results).

### 4.4. Lateral Flow Devices

**Figure 3:**
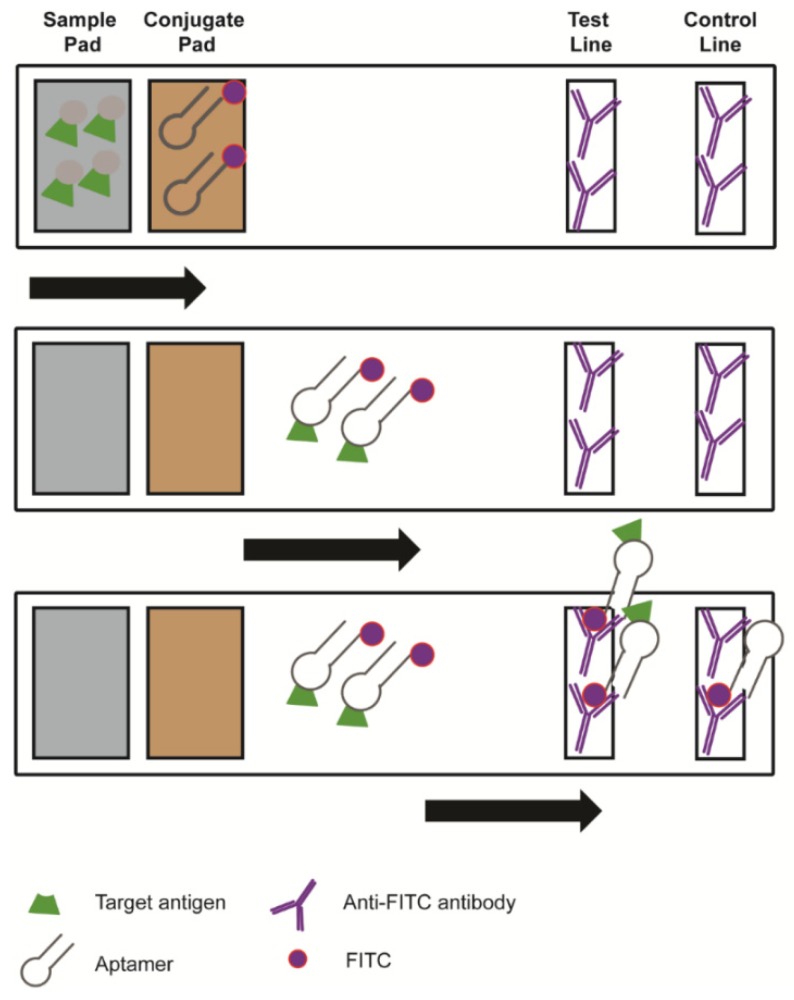
Schematic of a lateral flow device. A sample containing the target is added to the bottom of the test strip and, through capillary force, moves through the conjugate pad, where aptamer-reporter molecules attach to the target, to be captured by the antibodies in the test and control line.

Lateral flow devices (LFD) are utilised in both clinical laboratories and as point-of-care diagnostic devices. They represent a simple and rapid method for the detection of a number of pathogens and toxins (Figure 3). The general principle is that a ligand, such as an antibody, is labelled with a reporter molecule and immobilised within a testing zone on a porous membrane. The sample is then added to the bottom of the strip, and a buffer is typically added to the sample; migration towards the test strip occurs by capillary force. In addition to the target ligand, there is also a control ligand to ensure that the LFD is in proper working order [58,59].

Traditionally, antibodies have generally been the targeting ligand in LFDs. Given that aptamers can be selected to have a very high specificity (through the use of negative selection steps), cheap synthesis costs, and functional stability when stored at room temperature, there is the potential for a revolution in LFDs driven by aptamers. The high specificity of aptamers has already been exploited to develop aptamer-based LFD that can distinguish different strains of the influenza virus [60]. This device is based on a lateral flow design but pairs an antibody and an aptamer, the latter being very specific for a particular influenza strain. This dual recognition element lateral flow assay (DRELFA) can not only determine if a patient has the influenza virus, but can detect a specific virulent strain. This has important implications for future assays, considering that antibodies can demonstrate broad ranges of specificity but do not exhibit such an exquisite level of discrimination [60].

Overall, our aim in authoring this review has been to highlight ways in which aptamers can be usefully and seamlessly integrated into the clinical diagnostic space, as evidenced by a number of existing assays for which aptamers could replace antibodies with limited or no changes to protocols. However, there are many new and upcoming areas of development for which aptamers could potentially lead the charge in advancement. These would include rapid diagnostics using lateral flow devices [60], microfluidics [61], and biosensors [54,62]. Indeed, one area where aptamers could truly change practice and have an impact is in the diagnosis of bacterial strains, especially in the era of antimicrobial resistance.

Sepsis is caused by infection and is a major cause of death in newborns, children, and the elderly [63,64]. Due to the high mortality rates, it is recommended that treatment is initiated as soon as possible. Even a one hour delay can increase the risk of in-hospital mortality [63]. However, while the majority (>50%) of sepsis presentations are due to bacterial infections, up to 42% are culture negative, suggesting non-bacterial causes [65]. In addition, sepsis cases due to fungal organisms are rapidly increasing [66]. This is especially important, as increasing antimicrobial resistance necessitates the need to restrict the use of antibiotics [67]. Therefore, in order to identify the cause of sepsis correctly and to treat it effectively and efficiently, rapid diagnostics that can be used in routine practice in both developed and developing countries are required [68]. A number of aptamers have been generated to bacterial proteins [69,70], which could aide in the final diagnosis of infections, but would be unhelpful in the differential diagnosis of the cause of sepsis. An interesting development in the aptamer selection process, toggle SELEX [28], has allowed for the evolution of binding sequences that recognise a homologous region on multiple proteins. This can allow the development of broadly reactive aptamers, which is what Song et al. managed to isolate using six different bacterial strains [71]. Of note is that these six were a mixture of gram positive and gram negative bacteria, and that the aptamers showed similar binding affinities to each of them. These broadly reactive aptamers would remove the possible complexity of using aptamer cocktails in diagnostic assays [72,73]. A simple lateral flow device for the differential diagnosis of pathogenic agents in cases of sepsis could be utilised as a point of care device in any testing facility, including in remote and resource-poor communities without access to refrigerated supply-chains. However, for time-sensitive applications, such as sepsis management, the need for rapid diagnosis may also outweigh the semi-quantitative nature of LFD technology for use in well-equipped facilities. Additionally, recent advancements in LFD technology have improved quantitation capabilities and could offer additional advantages over current methods used in hospital testing facilities [70,74,75].

## 5. Conclusions

After reading this communication, you may be left asking “So if aptamers are so good, why haven’t they made the transition into the diagnostic arena?” Our response is “Good question, we ask ourselves the same thing almost daily.”

An editorial in 2010 asked this question and quite eloquently suggested that the issues relate to a lack of awareness, familiarity of antibodies, and the amount of money that has been spent on developing antibodies over the last 40 years or more [76]. As we approach the 30th anniversary of the first description of aptamers, is there hope that these ‘chemical antibodies’ will replace, or at least complement, conventional protein antibodies within the clinical setting? 

We feel it is important to note that monoclonal antibody reagents required almost 40 years of research and development to attain commercial success [20]; if aptamers are to follow the same path, there are still some 10 years of preliminary studies required to achieve the same starting point. The considerable financial investments made by many of the major pharmaceutical and biotech companies in the humanisation of monoclonal antibodies will likely impede the integration of aptamers into diagnostic assays, with familiarity of antibodies and commercial name brands driving much of the resistance against the adoption of aptamers as molecular probes for clinical diagnostic applications [20]. As a result, even after almost three decades in the development phase, aptamers seem to be perpetually stuck as the reagents of the future. It is clear, however, that support is growing for a switch to renewable, sequence-defined affinity reagents, with momentum gathering. This is evidenced by antibody validation standards attracting the attention of publishers and funding bodies [6,77]. With determination, hard work, and a bit of luck, the future may finally catch up to aptamer technology.

There are currently a number of companies that specialise in aptamers [78], with some listing a large catalogue of aptamers for R & D purposes. The majority of these aptamers have been validated in typical diagnostic applications, such as flow cytometry, immunohistochemistry and imaging, Western blot, and ELASA, though none have yet reached the market for use in diagnostic applications. One reason for this could be that the majority of aptamers are generated in academic environments where there are insufficient resources to further develop them for use in the clinical laboratory [79]. However, as aptamers use very similar or more simplified protocols compared to protein antibodies, as well as the same equipment already in place in clinical laboratories, we see it that the only limiting factor may well be the financial resources required to drive the next step in the aptamer revolution. Indeed, the number of publications in the area of clinical development for aptamers has been showing a steep increase over the last ten years. Perhaps aptamers are finally gaining their foothold in the diagnostic arena, due in large part to their uniqueness, as well as their superior specificity and sensitivity. The next step then, is to infiltrate other clinical arenas that have been reliant on antibodies for the past several decades or more.
